# Correction: Olaparib not cost-effective as maintenance therapy for platinum-sensitive, *BRCA1/2* germline-mutated metastatic pancreatic cancer

**DOI:** 10.1371/journal.pone.0315976

**Published:** 2024-12-12

**Authors:** Tarun Mehra, Judith E. Lupatsch, Thibaud Koessler, Konstantin Dedes, Alexander Reinhard Siebenhüner, Roger von Moos, Andreas Wicki, Matthias E. Schwenkglenks

The third author’s name is spelled incorrectly. The correct name is: Thibaud Koessler. The correct citation is: Mehra T, Lupatsch JE, Koessler T, Dedes K, Siebenhüner AR, von Moos R, et al. (2024) Olaparib not cost-effective as maintenance therapy for platinum-sensitive, BRCA1/2 germline-mutated metastatic pancreatic cancer. PLoS ONE 19(4): e0301271. https://doi.org/10.1371/journal.pone.0301271
