# Supplementary material for: Olaparib not cost-effective as maintenance therapy for platinum-sensitive, BRCA1/2 germline-mutated metastatic pancreatic cancer
Source: PLoS One. 2024 Apr 4;19(4):e0301271. doi: 10.1371/journal.pone.0301271 (PMC10994352; doi:10.1371/journal.pone.0301271)
Supplement: S1 File — (DOCX) [file pone.0301271.s001.docx]

# Supplement for

## Olaparib not cost-effective as maintenance therapy for platinum-sensitive, BRCA1/2 germline-mutated metastatic pancreatic cancer

**Authors**: Tarun Mehra^1¶*^, Judith E. Lupatsch^2¶^, Thibaud Kössler^3^, Konstantin Dedes^4^, Alexander Reinhard Siebenhüner^5^, Roger von Moos^6^, Andreas Wicki^7&^, Matthias E. Schwenkglenks^8&^

¶ These authors contributed equally

& These authors also contributed equally

*Corresponding author:

1) Dr. med. Tarun Mehra

Klinik für Medizinische Onkologie und Hämatologie

Universitätsspital Zürich

Rämistrasse 100

8091 Zürich

[tarun.mehra@usz.ch](mailto:tarun.mehra@usz.ch)

## Supplementary Methods S1

Population

The modelled target population reflected the POLO trial eligibility criteria [6], i.e. patients had *BRCA1/2*-germline-mutated metastatic pancreatic adenocarcinoma that had not progressed (i.e., patients had at least stable disease) after 16 weeks of first-line platinum-based chemotherapy with FOLFIRINOX (1). We assumed that patients were treated with FOLFIRINOX and not gemcitabine/nab-paclitaxel as FOLFIRINOX is the standard platinum-based first-line therapy and the design of the POLO trial had recruited patients with at least stable disease after platinum-based first-line chemotherapy.

Intervention, comparators and outcomes

In the intervention strategy, patients were assumed to receive two 150 mg olaparib tablets twice daily until progression, or serious adverse events warranting a treatment stop, or death. Analogically in the maintenance comparator strategy, patients were assumed to receive FOLRIRI until progression, or serious adverse events warranting a treatment stop, or death. The choice of FOLFIRI maintenance was based on the PRODIGE 35-PANOPTIMOX trial (2), published data by Chevalier et al (3), and expert opinion. The watch-and-wait comparator strategy postulated watch-and-wait until progression or death (4). All three strategies assumed that surviving patients would subsequently receive further-line chemotherapy with gemcitabine and nab-paclitaxel (5) and six weeks of palliative care were assumed at the end-of-life (6, 7). A flow-chart of the patient pathway assumed in our analysis is depicted in Figure 1, starting with the diagnosis of metastatic pancreatic cancer. We calculated the incremental cost-effectiveness ratio (ICER) comparing all three strategies using the olaparib strategy as main comparator. As they would not affect the cost-effectiveness and budget impact of the compared strategies, the costs of the first-line treatment phase including standard diagnostic work-up and 16 weeks of FOLFIRINOX chemotherapy were only considered in a per-patient comparison of treatment pathway costs.

Clinical input parameters

The progression free survival (PFS) PFS curves from the olaparib and placebo arms of the POLO trial were used to represent PFS in the olaparib strategy and comparator strategies, respectively. In the absence of a chemotherapy maintenance arm in the trial, to evaluate chemotherapy maintenance we used the PFS curve from the POLO placebo arm. This implies the comparison between the chemotherapy maintenance and watch-and-wait strategies is only based on cost differences. As the olaparib OS curve in the POLO trial does not differ significantly from the placebo OS curve, only the placebo OS curve was assumed to apply for all three strategies, in the base case. Kaplan-Meier plots were digitalized and an underlying time-to-event dataset estimated, as a basis for fitting suitable survival distributions. These curves were extrapolated for up to 10 years. The final selection of distributions (from candidates including exponential, lognormal, loglogistic, Weibull, Gompertz, generalised gamma) was based on the Akaike information criterion, comparison of overall survival at 5 and 10 years with that in the SEER database (8), and visual inspection. This led to the selection of a lognormal function for olaparib PFS and a log-logistic function for comparator PFS and OS curves (Suppl. Figure S1, suppl. Figure S2).

Utilities

We used published utilities for patients with metastatic pancreatic cancer with stable disease and post progression, as EQ-5D values from the POLO trial were not available. In the base case analysis, the utilities reported by Romanus et al. (9) were used for all strategies, and those reported by Gharaibeh et al. (10) in a scenario analysis. As disutility due to treatment-emergent adverse events is in general captured in utilities, it was not additionally considered in the base case, but in a scenario.

Medical resource use

Based on clinical practice we stipulated that patients would visit their oncologist monthly while on olaparib, every two weeks with FOLFIRI maintenance treatment and also monthly in the watch-and-wait phase, as well as every two weeks after transitioning to a further line of therapy. We further assumed a CT scan of the thorax and abdomen would be performed on average every 2 months, with an additional CT scan preceding each transition into a subsequent phase of the patient pathway.

We assumed that maintenance treatment would be administered for the published duration and that patients would switch to a next line of treatment at the time point of progression.

We estimated that screening the whole potentially eligible population for *BRCA1/2* germline mutations (Table S1) would require to screen a total of 579 patients per year. Considering costs for testing and genetic counseling, this would lead to a total amount of 98,814 CHF per year; 25 patients would need to be screened for one positive test.

We modelled CTCAE ≥ grade 3 adverse events in all three strategies, based on the literature as follows: CTCAE ≥ grade 3 events under olaparib treatment were assumed to occur in 40% of patients, based on an observed incidence of 39.6 events per 100 patients and in 23% of patients with watch-and-wait strategy based on the incidence of 23.3 events per 100 patients in the placebo arm of the POLO trial (4). CTCAE ≥ grade 3 events under FOLFIRI maintenance treatment were taken from a retrospective second line FOLFIRI study by Gebbia et al. (11), as the study by Chevalier et al., which reported AEs CTCAE ≥ grade 3 of 41%, did not differentiate the individual AEs sufficiently. We also modeled a rate of CTCAE ≥ grade 3 events of 70% for the next line of chemotherapy (5). Corresponding costs were added when state membership changed from PFS to PD. As information on hospitalizations for adverse events was not available for all studies, we based hospitalization for each individual AE on severity according to CTCAE version 5.0 and expert opinion. The adverse events are depicted in detail in Supplementary Table S3.

For FOLFIRINOX (relevant for the budget impact part), we extrapolated the rate of CTCAE ≥ grade 3 adverse events under FOLFIRINOX from the PRODIGE-24 trial (75.9%) (12) as the required information was not available for the ACCORD trial (1). We also assumed that all patients would follow through with the first line of FOLFIRINOX chemotherapy.

Unit costs

The cost of olaparib was retrieved from the official Swiss price list for outpatient drugs (13). The costs for FOLFIRINOX (without G-CSF support), FOLFIRI maintenance and gemcitabine/nab-paclitaxel chemotherapy are based on the official TARMED and medication cost lists (13, 14). Costs of consumables and staff were calculated by current standard of the cantonal hospital of Basel Land. The cost of somatic next-generation sequencing for *BRCA1/2* mutation status was retrieved from the Swiss official pricing list of diagnostic procedures (15). Costs for regular follow-up, diagnostic procedures, management of CTCAE ≥ grade 3 adverse events and the treatment of recurrent disease were based on real-world hospital billing in accordance with current versions of the Swiss outpatient tariff TARMED 1.09 (14), the diagnoses-related group (DRG)-based inpatient tariff SwissDRG (16), and the Swiss price list for diagnostic procedures (15). We assumed inpatient costs to be limited to the basic flat reimbursement fee per DRG without extra charges. We calculated the costs for 1.5 months of end of life care according to the figures published by Reich et al. (17), employing a correction factor for palliative care for oncological patients of 2.33 based on Panczac et al. (18), which we then inflated at an annual rate of 3.5% for 10 years to obtain contemporary prices for the cost year 2020 (19). Unit costs were calculated in Swiss francs (CHF). To facilitate international comparisons, costs in United States Dollars (USD) are additionally provided, based on a conversion rate of USD 1.10 per CHF 1 (2020 approximation). The unit costs used in the modelling are provided in Suppl. Table 3.

Epidemiological parameters

Epidemiological input parameters for the budget impact model are summarized in Supplementary Table 1. Switzerland has a population of 8.6 million (20), comparable in size to New York City, New Jersey or Virginia. The incidence of pancreatic cancer in Switzerland is 0.0185% per year (21), comparable to that in the United States (22). For our analysis we postulated a rate of metastatic disease of 52% (22), a disease control rate after platinum-based first-line chemotherapy of 70% (1) and a germline *BRCA1/2* mutation rate of 4% (23). The size of the total Swiss population as well as pancreatic cancer incidence were assumed to remain constant for the considered time period. The incidence was assumed to be uniformly distributed over time.

Partitioned survival model

A partitioned survival model (PSM) with *N* states calculates the probability patients being in various health states at a specific time during treatment with a particular therapy. State membership is determined by survival curves that do not overlap; in the case of an N-state model, it necessitates *N*-1 survival curves.

The cumulative survival function is

*S_n_*(*t*)

It describes the probability of a patient's survival to health state *n* or to a state with a lower index beyond a specified time *t*. The likelihood of a patient being in health state 1 is represented by *S*_1_*(t)*. Membership in health states 2,…,*n* −1 is determined by the difference between *S_n_*(*t*) and *S_n_*_−1_(*t*). The final health state *N* (when patients are dead) is 1−*S_N_*_−1_*(t*) (24).

Scenario and sensitivity analyses

Uncertainty analyses in the cost-effectiveness part included several scenarios, univariate sensitivity analysis and multivariate probabilistic sensitivity analysis (PSA). Utilities and probabilities were varied according to their standard error or by ±10% if standard errors were not available, costs by ±25%. We used beta distributions for utilities and probabilities and gamma distributions for costs, normal distributions for the standard errors of the lognormal and log-logistic survival functions. The outputs of 1,000 PSA iterations were used to generate cost-effectiveness scatterplots. The full set of uncertainty analyses was performed for the watch-and-wait comparator strategy as in the POLO-trial. We considered the following scenarios: (1) a potential price reduction of olaparib of 25%, (2) modelling the survival of the olaparib cohort with the olaparib OS curve from the POLO trial instead of the placebo OS curve, (3) using the same utility for the PDhealth state as a previous cost-effectiveness publication (25), (4) scenarios (2) and (3) combined, (4) a potential disutility for experiencing AEs of minus 0.2, (5) a potential PFS benefit for chemotherapy maintenance compared to watch & wait and *BRCA* panel testing potentially available for CHF 200 CHF.

Given that the POLO trial found no significant differences in OS, the base case analysis assumed that the modelling of survival could solely be based on the OS curve of the comparator arm, for both strategies. Subsequent scenario analyses, however, used the observed OS curves of both trial arms, which also did not lead to ICERs that could be considered cost-effective.

For the budget impact analyses, we performed scenario analyses by varying the *BRCA 1/2* germline mutation rate, with calculations performed with the assumption of a 2% or 7% mutation rate. We also performed the analysis with assumed disease control rates of 60% or 50%, respectively. We performed further scenario analysis by calculating the budget impact for the assumption of a price reduction in the germline next generation sequencing of *BRCA 1/2* to from CHF 3,329 (USD 3,662; base case) to CHF 200 (USD 220) as well as assuming a price reduction for olaparib of 25%.

Technical implementation and validation

The cost-effectiveness model was implemented in TreeAge Healthcare® (TreeAge Software, LLC, Williamstown, Massachusetts, USA (26)) and the budget impact model in Microsoft Excel®. We used DigitizeIt software (27) to digitalize published Kaplan-Meier curves and the R code from Guyot et al. (28) to estimate potential underlying individual patient data. All survival analyses were done in R.4.2.0 with the flexsurvreg packages. The TreeAge model calculation steps were validated in Excel or in R.

No artificial-intelligence based large language models were used in the writing of this manuscript.

Data-sharing statement

The analysis were performed using publically available data

## Supplementary Analysis

**Comparison of individual costs per treatment pathway**

With 5-year treatment costs of CHF 172,281 (USD 189,509) for the olaparib maintenance strategy on an individual basis accounting for patient attrition, FOLFIRI maintenance strategy per patient amounted to 88% and the watch and wait strategy to 73% of the cost (including first-line treatment and baseline diagnostics). The results can be found in detail in Supplementary Table S6, those of the scenario analysis in Supplementary Table S7.

The results of the 5-year per patient analysis reflected the budget impact results. Indeed, on a pure per-patient basis, the largest cost reductions were seen in the scenario analysis where the price of olabarib was reduced by 25%. In this sceneario, on a per patient basis, the savings potentiall achieved by a 25% price reduction in the olaparib drug price amounted to 6-fold the potential savings wehich could be achieved if of a reduction in germline *BRCA 1/2* screening costs were reduced to 200 CHF per analysis. However, if the per-patient costs were inflated for the costs of *BRCA 1/2* screening for the entire potentially eligible population, the relationship was reversed: savings estimated by the reduction in germline *BRCA* testing amounted to 4-fold the amount potentially saved by a 25% price reduction of olaparib.

## Supplementary Tables

**Supplementary Table S1. Abbreviations**

| **Abbreviation** | **Legend** |
| --- | --- |
| BRCA1/2 | Breast cancer 1 and 2, early-onset |
| CHF | Swiss Francs |
| gBRCA1/2 | germline Breast cancer 1 and 2, early-onset |
| HRR | homologous recombination repair |
| ICER | incremental cost-effectiveness-ratio |
| mPDAC | metastatic pancreatic ductal adeno-carcinoma |
| OS | overall survical |
| PARP | poly-ADP-ribose-polymerase |
| PD | progressive disease |
| PFS | progression-free survival |
| QALY | quality-adjusted life-year |
| USD | United States dollars |
| WTP | willingness-to-pay |

**Supplementary Table S2. Epidemiologic input parameters**

| **Parameter** | **Value** | **Reference** |
| --- | --- | --- |
| Swiss population | 8’606’000 | 2019 census (BfS) (20) |
| Incidence of pancreatic cancer (CH) | 0.0185% | NICER (21) |
| % Stage IV (metastatic disease) | 52% | SEER (22) |
| % controlled disease after 1^st^ line platinum-based chemotherapy | 70% | Conroy et all. (1) |
| % germline BRCA1/2 mutation | 4% | Waddell et all. (23) |
| Incidence baseline population p.a. ^1^ | 1592 | calculation |
| Incidence target population p.a. ^2^ | 23 | calculation |

Controlled disease: complete response, partial response or stable disease

^1^ all patients with pancreatic cancer in Switzerland

^2^ all patients with metastatic pancreatic cancer in Switzerland, controlled disease after 1^st^ line platinum-based therapy and *BRCA1/2* germline mutation

BfS: Bundesamt für Statistik (Switzerland)

**Supplementary Table S3. Clinical input parameters**

| **Parameter** | **Value** | **Reference** |
| --- | --- | --- |
| 1^st^ line FOLFIRINOX   - median treatment duration - rate of AE CTCAE ≥ grade 3 | - *6 months* - 75.9 % | - estimation, 6 4-week cycles - PRODIGE4/ACCORD11 (1) |
| Olaparib maintenance   - median treatment duration - median PFS - rate of AE CTCAE ≥ grade 3 | - 6 months - 7.4 months - 40% | - POLO (4) - POLO (4) - POLO (4) |
| FOLFIRI maintenance   - median treatment duration - median PFS - rate of AE CTCAE ≥ grade 3 | - 3.3 months - *4.5 months* - 70% | - PRODIGE 35-PANOPTIMOX (2) - estimation - Gebbia et al. (11) |
| Watch and wait maintenance   - median time to progression (PFS) - rate of AE CTCAE ≥ grade 3 | - 3.8 months - 23% | - POLO (4) - POLO (4) |
| 2^nd^ line gemcitabine & nab-paclitaxel   - median treatment duration - median time to progression (PFS) - rate of AE CTCAE ≥ grade 3 | - *3 months* - 3.8 months - 70% | - estimated - Mita et al. (5) - Mita et al. (5) |
| End of life care   - median time to death | - 1.5 months | - Osta et al. (7) |

PFS: progression free survival. Estimated values/assumptions are in italic

**Supplementary Table S4. Adverse events and costs**

|  | **Frequency %** | | | | **Ratio inpatient : outpatient (%)** | **Costs** | | | |
| --- | --- | --- | --- | --- | --- | --- | --- | --- | --- |
| **Adverse Event** | **Olaparib*** | **FOLFIRI Maintenance**** | **Watch-And-Wait (i.e. Placebo)*** | **2nd line Gemcitabine/nab-Paclitaxel******* |  | **Inpatient***** | | **Outpatient****** | |
| **Any** | 40 | 41****** | 23 | 70 | NA | NA | NA |  |  |
| **Fatigue/Asthenia** | 5 | NA | 2 | 0 | 20:80 | H61B | 6850 | 265 |  |
| **Nausea/Vomiting** | 1 | 7.5 | 4 | 0 | 80:20 | H61B | 6850 | 265 |  |
| **Anemia** | 11 | 7.5 | 3 | 26.7 | 20:80 | H61B | 6850 | 265 | 462.8 (red blood cell pack) |
| **Pain** | 2 | NA | 4 | NA | 50:50 | H61B | 6850 | 265 |  |
| **Diarrhea** | 0 | 15 | 0 | 3.3 | 80:20 | H61B | 6850 | 265 |  |
| **Decreased appetite/anorexia** | 3 | NA | 0 | 13.3 | 80:20 | H61B | 6850 | 265 |  |
| **Constipation** | 0 | NA | 0 | NA | 20:80 | H61B | 6850 | 265 |  |
| **Arthralgia** | 1 | NA | 0 | NA | 20:80 | H61B | 6850 | 265 |  |
| **Neutropenia** | NA | 17.5 | NA | 56.7^xx^ | 20:80 | Q60B | 8380 | 265 | 2 x 479.65 (Filgrastim) |
| **Thrombocytopenia** | NA | 2.5 | NA | 20 | 20:80 | H61B | 6850 | 265 | 1395 (platlet pack) |
| **Neuropathy** | NA | NA | NA | 13.3 | 0:100 | H61B | 6850 | 265 |  |
| **Alopecia** | NA | 10 | NA | 0 | 0:100 | H61B | 6850 | 265 |  |
| **Mucositis** | NA | 10 | NA | 0 | 80:20 | H61B | 6850 | 265 |  |
| **Hand-foot-syndrome** | NA | 0 | NA | NA | NA | H61B | 6850 | 265 |  |

Only adverse events CTCAE >= 3 considered. Only specifically mentioned AE considered for cost-effectiveness (CE) and business impact (BI) calculation. Totals are shown for descriptive purposes only. Individual AE with "NA" were set to the value "0" in the CE and BI calculations.

Legend:

| * POLO Trial |
| --- |
| ** Gebbia et al. 2010 |
| *** SwissDRG9.0 Abrechnungsversion. DRG, billing costs a multiple of the case weight by a base rate of 10'000 CHF without surcharges or deductions |
| **** Billing costs for an emergency hospital consultation (left) and relevant additional items according to expert opinion (right), in CHF |
| ***** Mita et al. 2019 |
| ****** Chevalier et al. 2020 |
| ^x^ includesperipheral sensory neuropathy and dysgeusia |
| ^xx^ includes Neutrpenia and febrile neutropenie |

**Supplementary Table S5. Cost input parameters**

| **Cost Item** | **Price (CHF)** | **Sensitivity** | **Reference** |
| --- | --- | --- | --- |
| Cost histology | 519.20 CHF | 25% (gamma) | Billing cost, Cantonal Hospital of Liestal, CH |
| Cost NGS somatic (full cost) | 2’272.65 CHF | 25% (gamma) | Billing cost, Cantonal Hospital of Liestal, CH |
| Cost NGS germline (full cost) | 3’329.49 CHF | 25% (gamma) | Billing cost, University Hospital of Lausanne, CH (official Swiss listing price) |
| Cost Biopsy | 970.35 CHF | 25% (gamma) | Billing cost, Cantonal Hospital of Liestal, CH |
| Cost genetic counselling | 596.27 CHF | 25% (gamma) | Billing cost, University Hospital of Lausanne, CH (official Swiss listing price) |
| Cost computer tomography thorax/abdomen with contrast medium (full cost incl. visit) | 737.32 CHF | 25% (gamma) | Billing cost, Cantonal Hospital of Liestal, CH |
| Cost regular consultation (full cost) | 188 CHF | 25% (gamma) | Billing cost, Cantonal Hospital of Liestal, CH |
| Cost emergency consultation - outpatient | 265 CHF | 25% (gamma) | Billing cost, Cantonal Hospital of Liestal, CH |
| Cost Olaparib | 191.4 CHF / day | 25% (gamma) | Swiss listing price |
| Cost FOLFIRINOX | 2’035.5CHF |  | Billing cost, Cantonal Hospital of Liestal, CH |
| (cost per dose, 100%) |  |  |  |
| Cost Gemcitabine + nab-Paclitaxel | 1’333.5 CHF | 25% (gamma) | Billing cost, Cantonal Hospital of Liestal, CH |
| (cost per dose, 100%) |  |  |  |
| FOLFIRI maintenance | 1’682 CHF |  | Billing cost, Cantonal Hospital of Liestal, CH |
| (cost per dose, 80%) |  | 25% (gamma) |  |
| 1 cycle, 1cycle q2w |  |  |  |
| Cost GM-CSF/Filgrastim (non-pegylated, 30 mio UI)* | 485.75 CHF |  |  |
|  |  | 25% (gamma) | Billing cost, Cantonal Hospital of Liestal, CH |

| Cost hospitalization (DRG)* |  | 25% (gamma) | SwissDRG billing catalogue for 2020 |
| --- | --- | --- | --- |
| tumor progression/fatigue | 6’850 CHF |  |  |
| pain/nausea/vomiting/diarrhea | 6’850 CHF |  |  |
| febrile neutropenia | 8’380 CHF |  |  |
| pneumonia | 6’850 CHF |  |  |
| sepsis | 9’920 CHF |  |  |
| pulmonary embolism | 9’920 CHF |  |  |
|  |  |  |  |
|  |  |  |  |
|  |  |  |  |
| Cost palliative care (full cost for 1.5 months) | 14’500 CHF |  | References: |
|  |  | 25% (gamma) | -          Reich et al. (17) |
|  |  |  | -          Panczak et al. (18) |

* included in the DRG for inpatients, billed 2x in case of severe neutropenia not requiring hospitalisation

** assuming a baserate of 10’000 CHF.

**Supplementary Table S6.** **Estimation of 5-year treatment costs per patient, per treatment pathway**

**a** Olaparib maintenance

|  | **Olaparib** | | | | | | |
| --- | --- | --- | --- | --- | --- | --- | --- |
|  | Baseline | Year 1 | Year 2 | Year 3 | Year 4 | Year 5 | Total |
| Drug costs, first line (FOLFIRINOX 100% q2w 16W) | 16'284 | - | - | - | - | - | 16'284 |
| Costs of care* | 0 | 1'562 | 608 | 315 | 188 | 123 | 2'797 |
| Costs of side-effects* | 0 | 389 | 113 | 44 | 21 | 12 | 577.84 |
| Diagnostic costs* | 0 | 2'772 | 1'079 | 559 | 334 | 218 | 4'961 |
| Drug costs, maintenance* | 0 | 43'745 | 17'033 | 8'818 | 5'272 | 3'436 | 78'304 |
| Costs 2nd line* | 0 | 17'316 | 16'655 | 7'908 | 3'984 | 2'276 | 48'139 |
| Palliative care costs* | 0 | 4'509 | 5'123 | 2'323 | 1'046 | 530 | 13'531 |
| Cost biopsy | 970 | - | - | - | - | - | 970 |
| Cost histology | 519 | - | - | - | - | - | 519 |
| Cost NGS Somatic | 2'273 | - | - | - | - | - | 2'273 |
| Cost NGS Germline | 3'329 | - | - | - | - | - | 3'329 |
| Cost genetic counselling | 596 | - | - | - | - | - | 596. |
| Total costs | 23'971 | 70'293 | 40'611 | 19'966 | 10'846 | 6'594 | **172'281** |

**b** FOLFIRI maintenance

|  | **FOLFIRI maintenance** | | | | | | |
| --- | --- | --- | --- | --- | --- | --- | --- |
|  | Baseline | Year 1 | Year 2 | Year 3 | Year 4 | Year 5 | Total |
| Drug costs, first line (FOLFIRINOX 100% q2w 16W) | 16'284 | - | - | - | - | - | 16'284 |
| Costs of care* | 0 | 6'356 | 829 | 292 | 148 | 90 | 7'717 |
| Costs of side-effects* | 0 | 2'241 | 190 | 39 | 14 | 7 | 2'491 |
| Diagnostic costs* | 0 | 1'869 | 244 | 86 | 44 | 26 | 2'269 |
| Drug costs, maintenance* | 0 | 14'271 | 1'861 | 655 | 333 | 201 | 17'320 |
| Costs 2nd line* | 0 | 30'958 | 29'289 | 15'060 | 8'376 | 5'169 | 88'852 |
| Palliative care costs* | 0 | 4'509 | 5'123 | 2'323 | 1'046 | 530 | 13'531 |
| Cost biopsy | 970 | - | - | - | - | - | 970 |
| Cost histology | 519.20 | - | - | - | - | - | 519 |
| Cost NGS Somatic | 2'273 | - | - | - | - | - | 2'273 |
| Cost NGS Germline | - | - | - | - | - | - | - |
| Cost genetic counselling | - | - | - | - | - | - | - |
| Total costs | 20'046 | 60'207 | 37'535 | 18'454 | 9'961 | 6'023 | **152'226** |

**c** Watch & Wait maintenance

|  | **Watch and Wait** | | | | | | |
| --- | --- | --- | --- | --- | --- | --- | --- |
|  | Baseline | Year 1 | Year 2 | Year 3 | Year 4 | Year 5 | Total |
| Drug costs, first line (FOLFIRINOX 100% q2w 16W) | 16'284 | - | - | - | - | - | 16'284 |
| Costs of care* | 0 | 1'054 | 137 | 48 | 25 | 15 | 1'279 |
| Costs of side-effects* | 0 | 406 | 35 | 7 | 3 | 1 | 452 |
| Diagnostic costs* | 0 | 1'869 | 244 | 86 | 44 | 26 | 2'269 |
| Drug costs, maintenance* | 0 | - | - | - | - | - | - |
| Costs 2nd line* | 0 | 30'958 | 29'289 | 15'060 | 8'376 | 5'169 | 88'852 |
| Palliative care costs* | 0 | 4'509 | 5'123 | 2'323 | 1'046 | 530 | 13'531 |
| Cost biopsy | 970 | - | - | - | - | - | 970 |
| Cost histology | 519 | - | - | - | - | - | 519 |
| Cost NGS Somatic | 2'273 | - | - | - | - | - | 2'273 |
| Cost NGS Germline | - | - | - | - | - | - | - |
| Cost genetic counselling | - | - | - | - | - | - | - |
| Total costs | 20'046 | 38'797 | 34'827 | 17'524 | 9'493 | 5'741 | **126'429** |

All costs are depicted in Swiss francs (CHF)

Costs of care: includes visits to the outpatient clinic, routine blood samples. Costs of side-effects: includes costs for hospitalisations due to side effects, visits to the outpatient clinic due to side effects, etc. Diagnostic costs: regular imaging studies.

**Supplementary Table S7.** **Sensitivity analysis for 5-year per patient pathways**

**a** Estimated 5-year treatment costs per patient per clinical pathway *limiting BRCA 1/2* screening costs to the eligible population (N=23)

| Estimated treatment course cost per patient (CHF) | Olaparib | FOLFIRI | Watch & Wait |
| --- | --- | --- | --- |
| Base Case | 172'281 | 152'226 | 126'429 |
| NGS somatic 200 CHF | 170'208 | 150'153 | 124'356 |
| NGS germline 200 CHF | 169'152 | 152'226 | 126'429 |
| Somatic & germline 200 CHF | 167'079 | 150'153 | 124'356 |
| Olaparib 25% cost reduction | 152'705 | 152'226 | 126'429 |

**b** Estimated 5-year treatment costs per patient per clinical pathway including accrural of germline *BRCA 1/2* screening costs of negative screens for the potentially eligible population (N=579)

| Estimated treatment course cost per patient (CHF)* | Olaparib | FOLFIRI | Watch & Wait |
| --- | --- | --- | --- |
| Base Case | 267'170 | 152'226 | 126'429 |
| BRCA 2% | 357'750 | 152'226 | 126'429 |
| BRCA 7% | 223'788 | 152'226 | 126'429 |
| DCR 60% | 265'899 | 152'226 | 126'429 |
| DCR 50% | 263'948 | 152'226 | 126'429 |
| NGS somatic 200 CHF | 265'098 | 150'153 | 124'356 |
| NGS germline 200 CHF | 188'401 | 152'226 | 126'429 |
| Somatic & germline 200 CHF | 170'044 | 150'153 | 124'356 |
| Olaparib 25% cost reduction | 247'594 | 152'226 | 126'429 |

DCR: disease control rate; NGS: next generation sequencing;

NGS somatic 200 CHF: somatic NGS sequencing costs reduced from 2,272 CHF to 200 CHF; NGS germline 200 CHF: NGS costs for germline sequencing reduced from 3,329 CHF to 200 CHF; Somatic & germline 200 CHF: both NGS sequencing costs, somatic and germline, reduced to 200 CHF each; BRCA 2%: incidence of *BRCA 1/2* prevalence decreased from 4% to 2%; BRCA 7%: incidence of *BRCA 1/2* prevalence increased from 4% to 7%; DCR 60%: DCR reduced from 70% to 60%; DCR 50%: DCR reduced from 70% to 50%; Olaparib cost reduction: drug price reduction of olaparib by 25%

All costs in Swiss francs (CHF)

## Supplementary Figures

**Supplementary Fig S1. Fitted survival curves for PFS Kaplan Meier plots from POLO trial**


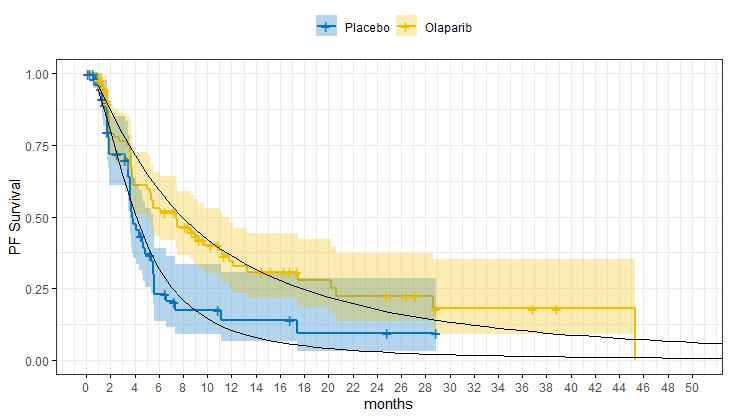


Figure notes: lognormal function for olaparib; log-logistic function for placebo, PFS: progression free

**Supplementary Fig S2. Fitted overall survival curves for overall survival Kaplan Meier plots from POLO trial**


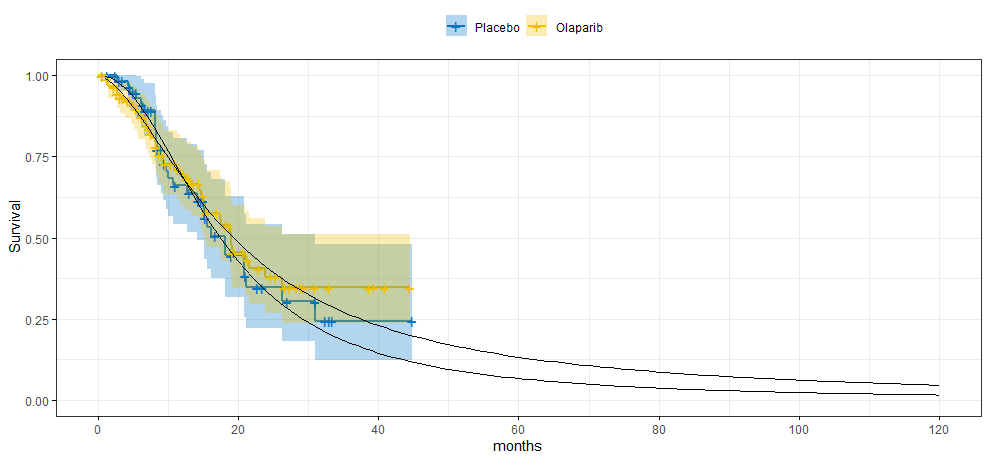


Figure notes: log-logistic function for olaparib and placebo

**Supplementary Fig S3. Tornado diagram – incremental net monetary benefit**


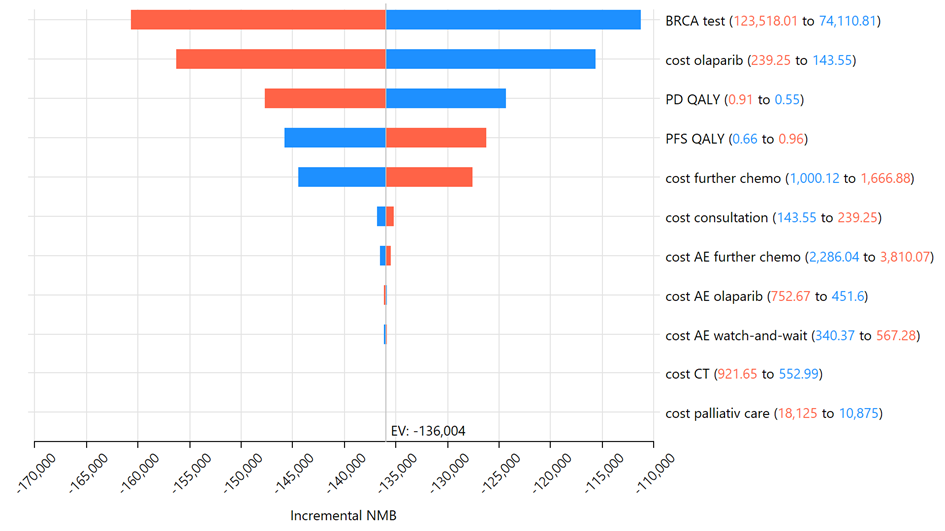


Figure note: Incremental net monetary benefit for olaparib strategy versus watch-and-wait strategy. The graph includes an EV line that reflects the base case ICER, assuming that each input is set to its best estimated value. Each bar on the graph is divided into a blue section, representing the input range from the lower bound to the base case value, and a red section, representing the input range from the base case value to the upper bound. PD: progressive disease, PFS: progression free survival, AE: adverse events. All costs are depicted in Swiss francs (CHF)

**Supplementary Fig S4.**


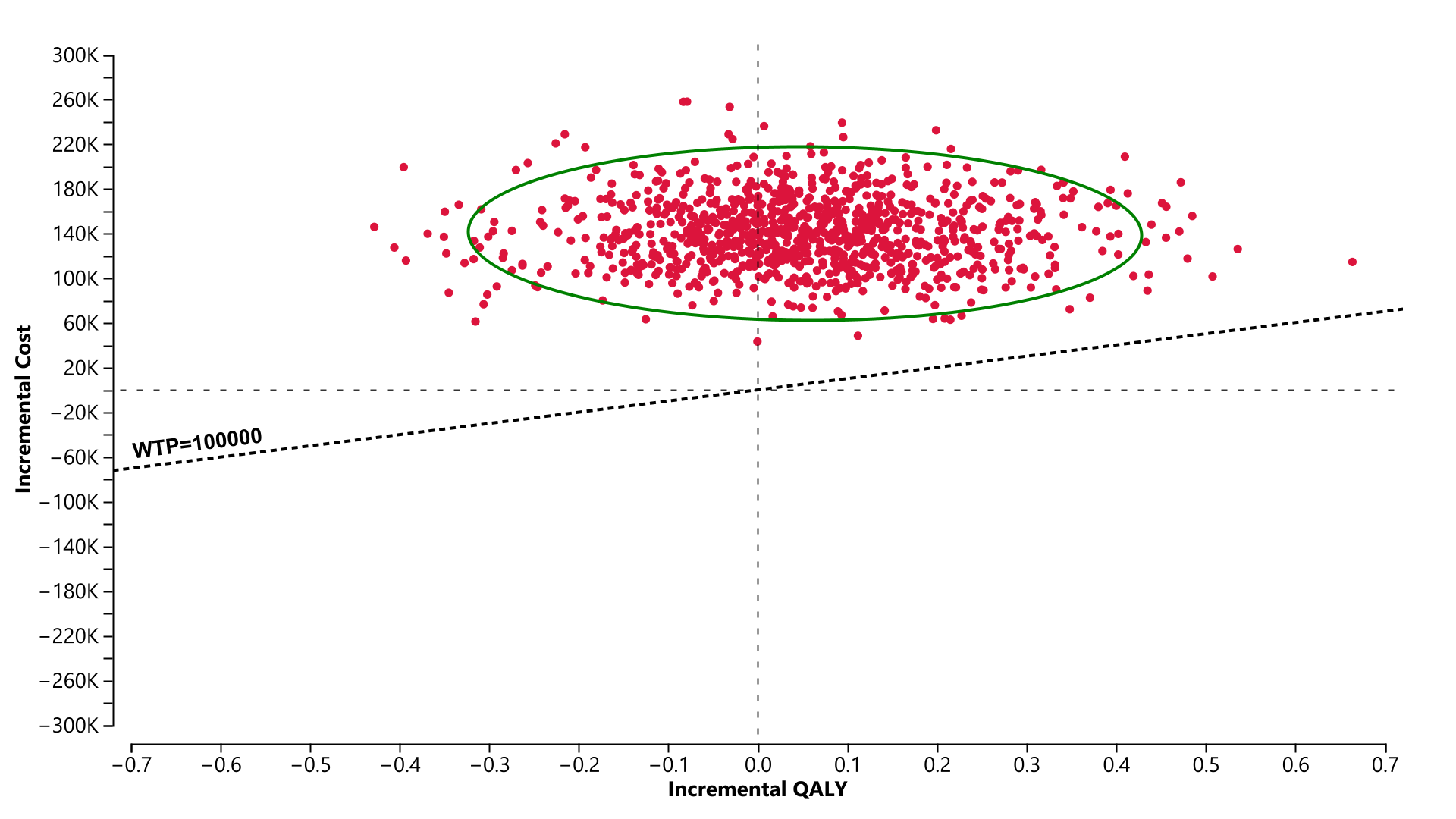


**a** Probabilistic Sensitivity Analysis Olaparib versus Observation – Basecase


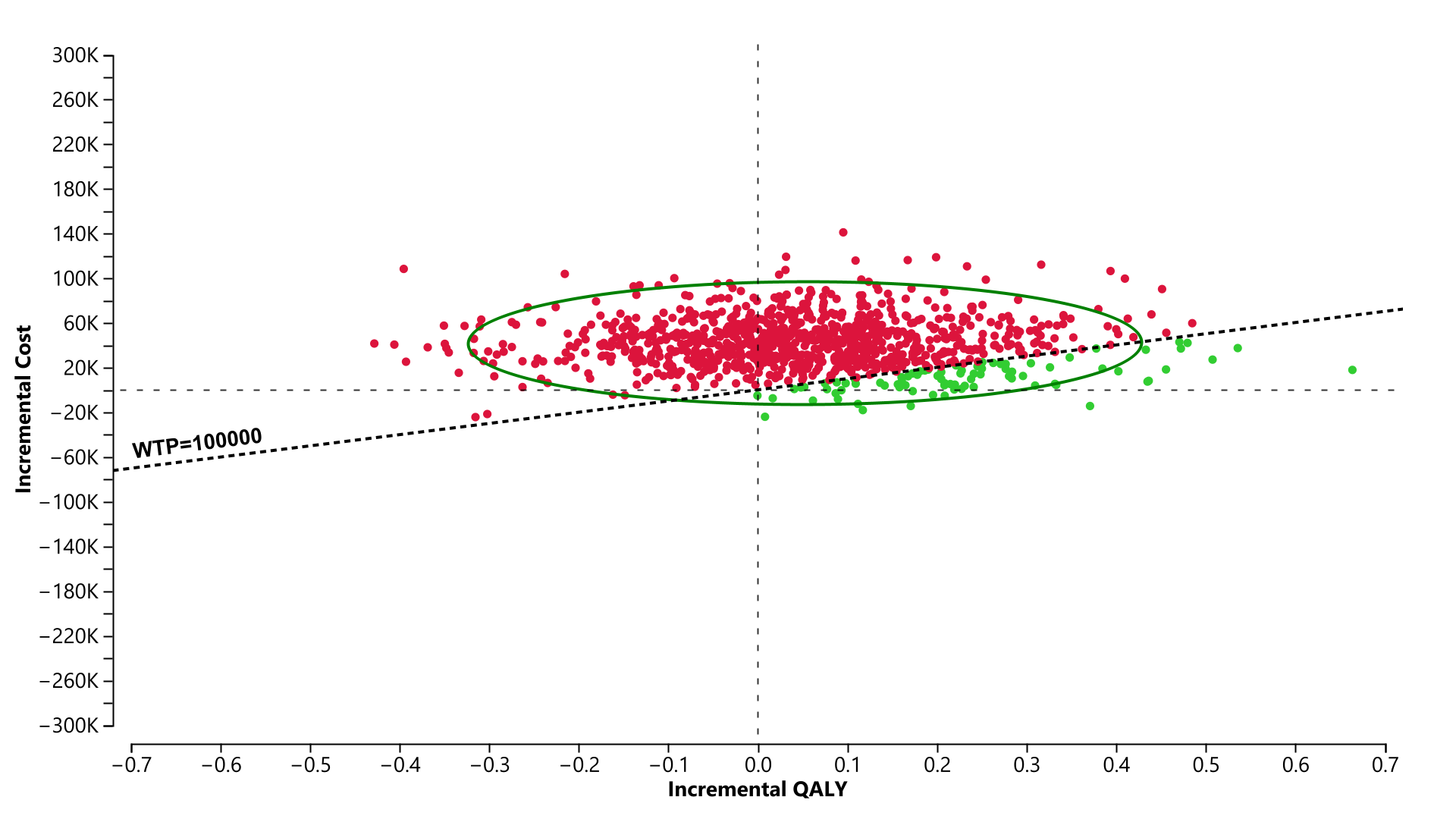


**b** Probabilistic Sensitivity Analysis Olaparib versus Observation – without cohort BRCA testing

Figure notes: WTP: willingness-to-pay threshold set to 100’000 Swiss Francs. QALYS: quality-adjusted life years. All costs are depicted in Swiss francs (CHF).

**Supplementary Figure S5**

5-year total costs for the treatment of platinum-sensitive germline *BRCA 1/2* mutated pancreatic cancer in Switzerland with either olaparib maintenance, FOLFIRI maintenance or watch and wait strategy, a) accounting for germline BRCA 1 / 2 screening costs and b) not accounting for germline *BRCA 1/2* screening costs for the screening of the entire cohort of potential eligible patients (eligible population of N=23 per year, potentially eligible population of N = 579 per year) .

**
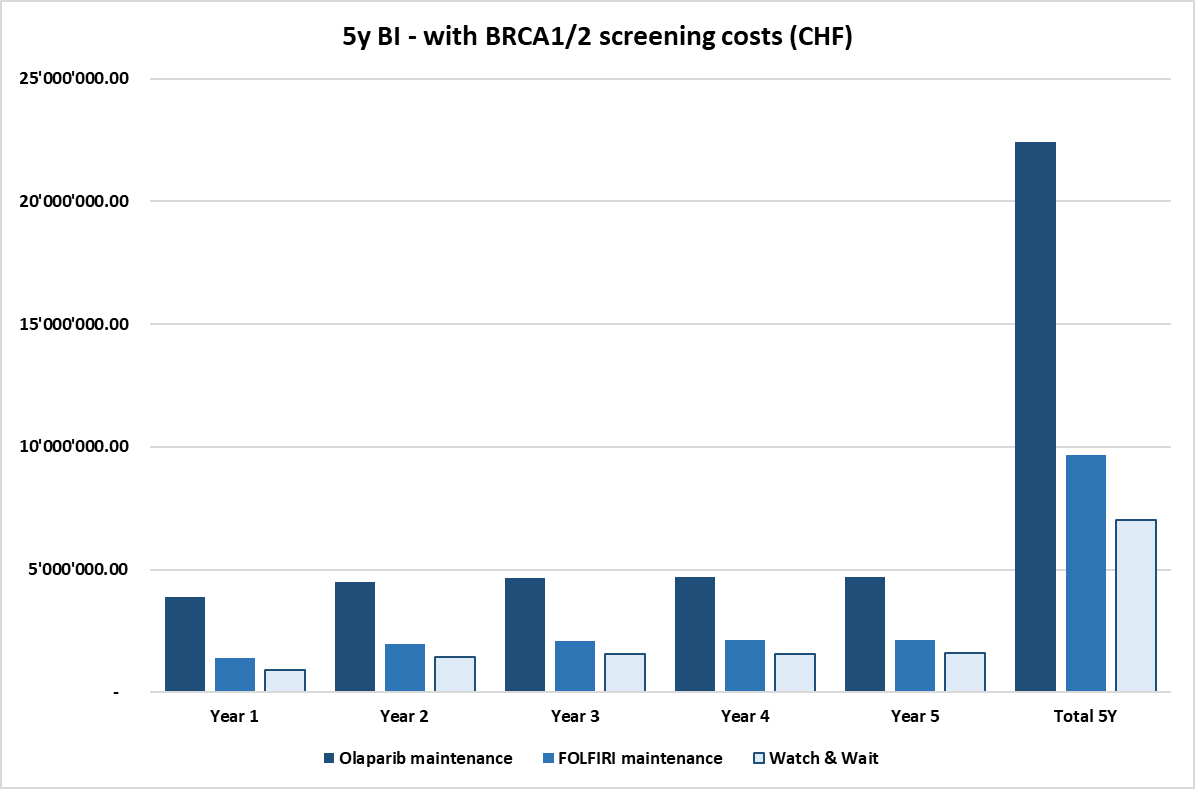
**

**Supplementary Figure S5a**


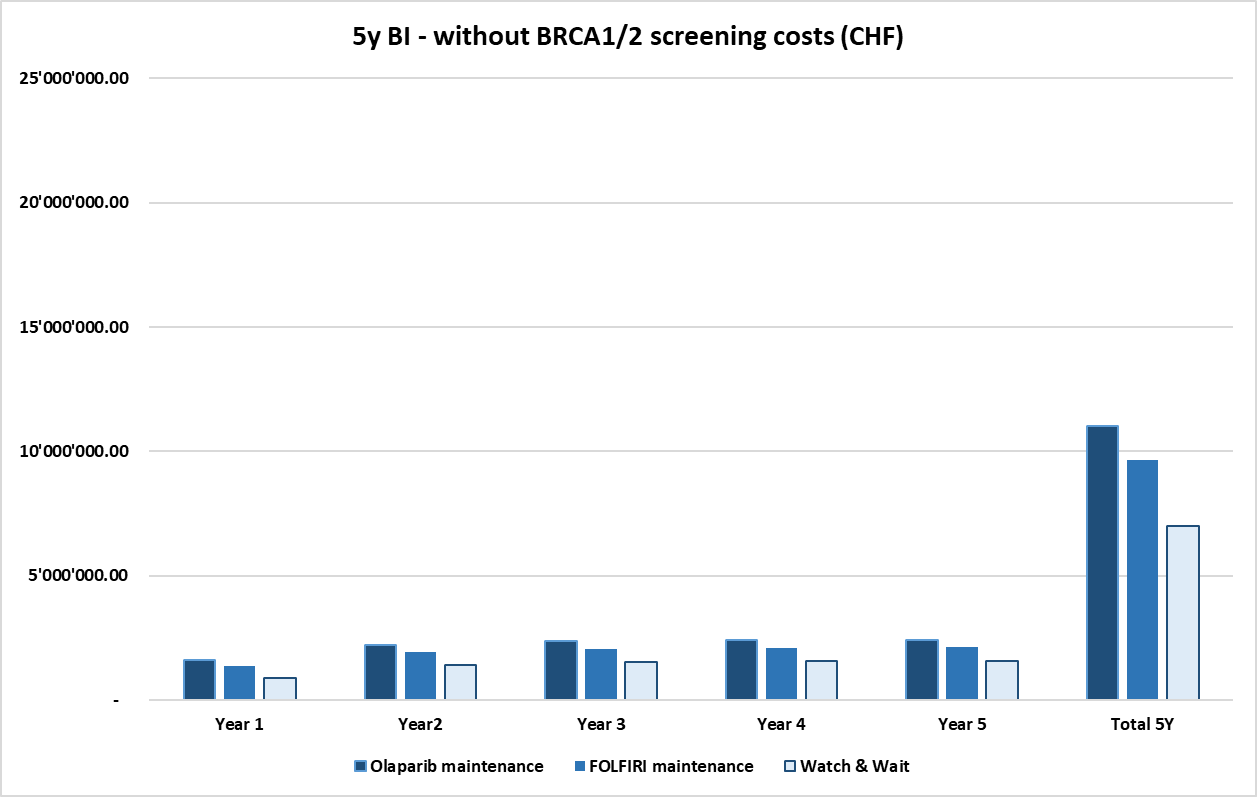


**Supplementary Figure S5b**

BI: Budget impact; CHF: Swiss francs

Costs include maintenance therapy, 2nd line chemotherapy with gemcitabine and nab-paclitaxel, as well as palliative end of life care, including costs of side-effects, routine medical visits and laboratory testing as well as imaging costs. Costs for standard care first line platinum-based chemotherapy and routine somatic genetic testing not included.

**Supplementary Figure S6**

Scenario analysis of the 5-year total costs for the treatment of platinum-sensitive germline *BRCA 1/2* mutated pancreatic cancer in Switzerland with either olaparib maintenance, FOLFIRI maintenance or watch and wait strategy (accounting for costs for germline *BRCA 1/2* companion diagnostics for screening failures) (yearly incidence of n=23).

**
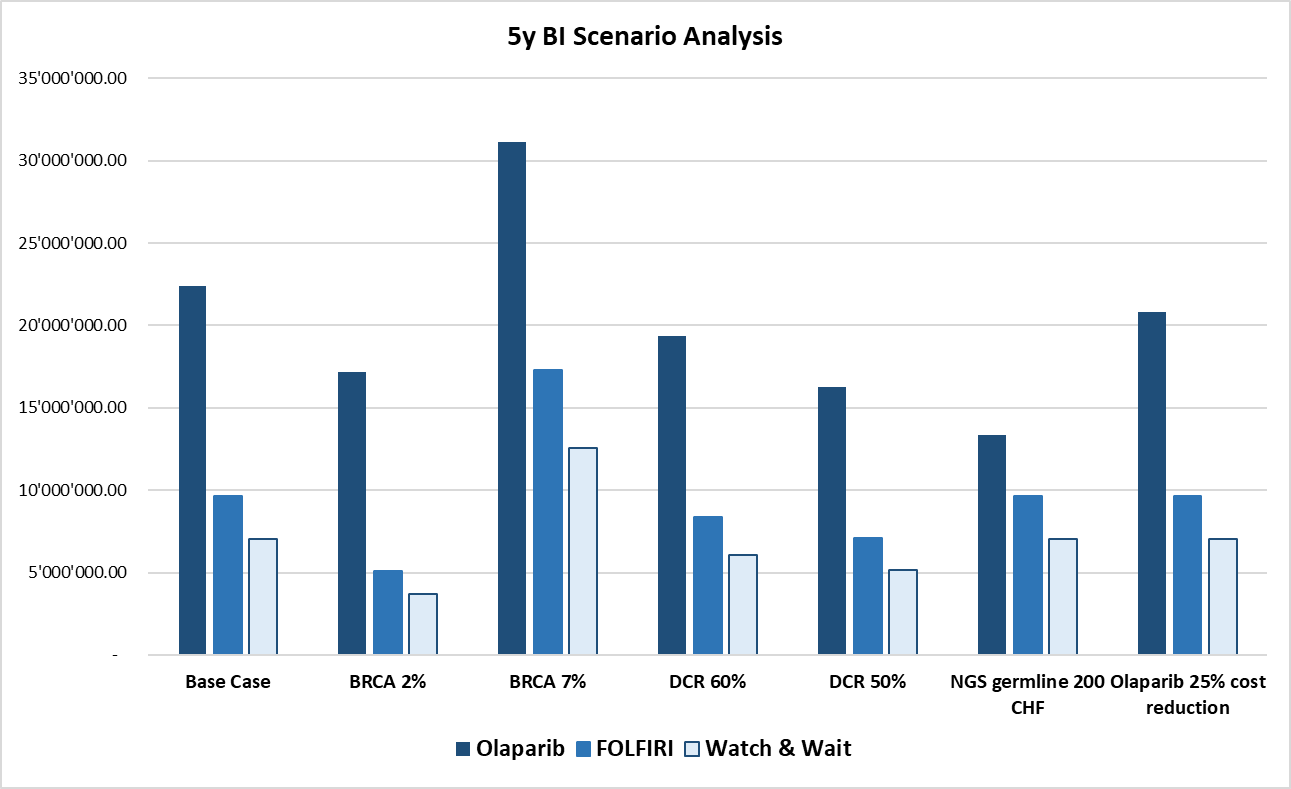
**

DCR: disease control rate; NGS: next generation sequencing. All costs in Swiss francs (CHF)

Costs include maintenance therapy, 2nd line chemotherapy with gemcitabine and nab-paclitaxel, as well as palliative end of life care, including costs of side-effects, routine medical visits and laboratory testing as well as imaging costs. Costs for standard care first line platinum-based chemotherapy and routine somatic genetic testing not included.

## Supplementary References

1. Conroy T, Desseigne F, Ychou M, Bouche O, Guimbaud R, Becouarn Y, et al. FOLFIRINOX versus gemcitabine for metastatic pancreatic cancer. N Engl J Med. 2011;364(19):1817-25.

2. Laetitia Dahan JMP KLM, Nicolas Williet, Jérôme Desrame, Julien Volet, Caroline Petorin, David Malka, Christine Rebischung, Thomas Aparicio, Cedric Lecaille, Yves Rinaldi, Anthony Turpin, Anne Laure Bignon, Jean-Baptiste Bachet, Jean Francois Seitz, Come Lepage, Eric Francois. FOLFIRINOX until progression, FOLFIRINOX with maintenance treatment, or sequential treatment with gemcitabine and FOLFIRI.3 for first-line treatment of metastatic pancreatic cancer: A randomized phase II trial (PRODIGE 35-PANOPTIMOX). SCO: Journal of Clinical Oncology. 2018:4000.

3. Chevalier H, Vienot A, Lièvre A, Edeline J, El Hajbi F, Peugniez C, et al. FOLFIRINOX De‐Escalation in Advanced Pancreatic Cancer: A Multicenter Real‐Life Study. The Oncologist. 2020;25(11):e1701-e10.

4. Golan T, Hammel P, Reni M, Van Cutsem E, Macarulla T, Hall MJ, et al. Maintenance Olaparib for Germline BRCA-Mutated Metastatic Pancreatic Cancer. N Engl J Med. 2019;381(4):317-27.

5. Mita N, Iwashita T, Uemura S, Yoshida K, Iwasa Y, Ando N, et al. Second-Line Gemcitabine Plus Nab-Paclitaxel for Patients with Unresectable Advanced Pancreatic Cancer after First-Line FOLFIRINOX Failure. Journal of clinical medicine. 2019;8(6).

6. Bennett MI, Ziegler L, Allsop M, Daniel S, Hurlow A. What determines duration of palliative care before death for patients with advanced disease? A retrospective cohort study of community and hospital palliative care provision in a large UK city. BMJ Open. 2016;6(12):e012576.

7. Osta BE, Palmer JL, Paraskevopoulos T, Pei BL, Roberts LE, Poulter VA, et al. Interval between first palliative care consult and death in patients diagnosed with advanced cancer at a comprehensive cancer center. Journal of palliative medicine. 2008;11(1):51-7.

8. SEER. Cancer Stat Facts: Pancreatic Cancer: National Cancer Institute; [Available from: <https://seer.cancer.gov/statfacts/html/pancreas.html>.

9. Romanus D, Kindler HL, Archer L, Basch E, Niedzwiecki D, Weeks J, et al. Does health-related quality of life improve for advanced pancreatic cancer patients who respond to gemcitabine? Analysis of a randomized phase III trial of the cancer and leukemia group B (CALGB 80303). J Pain Symptom Manage. 2012;43(2):205-17.

10. Gharaibeh M, McBride A, Alberts DS, Slack M, Erstad B, Alsaid N, et al. Economic Evaluation for USA of Systemic Chemotherapies as First-Line Treatment of Metastatic Pancreatic Cancer. Pharmacoeconomics. 2018;36(10):1273-84.

11. Gebbia V, Maiello E, Giuliani F, Borsellino N, Arcara C, Colucci G. Irinotecan plus bolus/infusional 5-Fluorouracil and leucovorin in patients with pretreated advanced pancreatic carcinoma: a multicenter experience of the Gruppo Oncologico Italia Meridionale. Am J Clin Oncol. 2010;33(5):461-4.

12. Conroy T, Hammel P, Hebbar M, Ben Abdelghani M, Wei AC, Raoul JL, et al. FOLFIRINOX or Gemcitabine as Adjuvant Therapy for Pancreatic Cancer. New Engl J Med. 2018;379(25):2395-406.

13. Gesundheit Bf. Spezialitätenliste (SL). Bern, Switzerland: Eidgenössisches Department des Innern EDI; 2020.

14. Gesuntheit Bf. Tarifsystem TARMED Version 1.09. Bern, Switzerland: Eidgenössisches Department des Innern EDI; 2018.

15. Gesundheit Bf. Analyseliste (AL). Bern, Switzerland: Eidgenössisches Departement des Inneren EDI; 2020.

16. SwissDRG. Fallpauschalenkatalog SwissDRG Abrechnungsversion 9.0 (2020/2020). Bern, Switzerland2019.

17. Reich O, Signorell A, Busato A. Place of death and health care utilization for people in the last 6 months of life in Switzerland: a retrospective analysis using administrative data. BMC Health Services Research. 2013;13(1):116.

18. Panczak R, Luta X, Maessen M, Stuck AE, Berlin C, Schmidlin K, et al. Regional Variation of Cost of Care in the Last 12 Months of Life in Switzerland: Small-area Analysis Using Insurance Claims Data. Med Care. 2017;55(2):155-63.

19. Fisher ES, Bynum JP, Skinner JS. Slowing the growth of health care costs--lessons from regional variation. The New England journal of medicine. 2009;360(9):849-52.

20. Bevölkerungsentwicklung im Jahr 2019: provisorische Ergebnisse. In: BfS BfS, editor. Neuchâtel, Switzerland2020.

21. Die Statistiken zur Nationalen Krebsinzidenz. In: NICER NK, editor. Zürich, Switzerland2020.

22. Society AC. Key Statistics for Pancreatic Cancer. 2021.

23. Waddell N, Pajic M, Patch AM, Chang DK, Kassahn KS, Bailey P, et al. Whole genomes redefine the mutational landscape of pancreatic cancer. Nature. 2015;518(7540):495-501.

24. Network TCRA. Partitioned survival models. 2024.

25. Wu B, Shi L. Cost-Effectiveness of Maintenance Olaparib for Germline BRCA-Mutated Metastatic Pancreatic Cancer. J Natl Compr Canc Netw. 2020;18(11):1528-36.

26. LLC TS. Version 2022 ed. Williamstown, Massachusets, USA.

27. GmbH DR. Digizelt. 2019.

28. Guyot P, Ades AE, Ouwens MJ, Welton NJ. Enhanced secondary analysis of survival data: reconstructing the data from published Kaplan-Meier survival curves. BMC Med Res Methodol. 2012;12:9.
