# Supplementary material for: Olaparib not cost-effective as maintenance therapy for platinum-sensitive, BRCA1/2 germline-mutated metastatic pancreatic cancer
Source: PLoS One. 2024 Apr 4;19(4):e0301271. doi: 10.1371/journal.pone.0301271 (PMC10994352; doi:10.1371/journal.pone.0301271)
Supplement: S2 File — (PDF) [file pone.0301271.s002.pdf]

ESMO-MCBS

ESMO-Magnitude of Clinical Benefit Scale

ESMO-MAGNITUDE OF CLINICAL BENEFIT SCALE V1.1 EVALUATION FORM 2B

For therapies that are not likely to be curative with primary endpoint PFS

Name of study:	POLO Trial				
Study medicine:	Olaparib	Indication:	gL BRCA 1/2 mut. pl-sens. mPAC*		
First author:	Talia Golan, M.D.	Year:	2019	Journal:	NEJM
Name of evaluator:	Tarun Mehra M.D.				

* germline BRCA 1/2 mutated platinum-sensitive metastatic adenocarcinoma of the pancreas

If median PFS with standard treatment >6 months

GRADE 3	HR ≤0.65 AND gain ≥3 months	✓
GRADE 2	HR ≤0.65 BUT gain <3 months	
GRADE 1	HR >0.65	

Mark with √ if relevant

	3	2	1
**Preliminary magnitude of clinical benefit grade (highest grade scored)**	✓		

Non-curative setting grading - 5 and 4 indicates a substantial magnitude of clinical benefit

HR, hazard ratio; PFS, progression-free survival

ESMO-MCBS

ESMO-Magnitude of Clinical Benefit Scale

Early stopping or crossover

Did the study have an early stopping rule based on interim analysis of survival?	no	◯
Was the randomization terminated early based on the detection of overall survival advantage at interim analysis?	no	◯

If the answer to both is “yes”, then see letter “E” in the adjustment section below — Mark with √ if relevant

Toxicity assessment

Is the new treatment associated with a statistically significant incremental rate of:

«Toxic» death >2%	no	◯
Cardiovascular ischemia >2%	no	◯
Hospitalisation for «toxicity» >10%	no	◯
Excess rate of severe CHF >4%	no	◯
Grade 3 neurotoxicity >10%	no	◯
Severe other irreversible or long lasting toxicity >2% please specify:	no	◯

(Incremental rate refers to the comparison versus standard therapy in the control arm) — Mark with √ if relevant

Quality of life/Grade 3-4 toxicities* assessment

Was QoL eveluated as secondary outcome?		✓
Does secondary endpoint QoL show improvement?	no	◯
Are there statistically significantly less grade 3-4 toxicities impacting on daily well-being?*	no	◯

*This does not include alopecia, myelosuppression, but rather chronic nausea, diarrhoea, fatigue, etc. — Mark with √ if relevant

CHF, congestive heart failure; QoL, quality of Life

Adjustments

A When OS as secondary endpoint shows improvement, it will prevail and the new scoring will be done according to form 2a. No improvement (Kindler et al. 2022)

B Downgrade 1 level if there is one or more of the above incremental toxicities associated with the new medicine. No

C Downgrade 1 level if the medicine ONLY leads to improved PFS (mature data shows no OS advantage) and QoL assessment does not demonstrate improved QoL. True

D Upgrade 1 level if improved QoL or if less grade 3-4 toxicities that bother patients are demonstrated. No

E Upgrade 1 level if study had early crossover because of early stopping or crossover based on detection of survival advantage at interim analysis. No

F Upgrade 1 level if there is a long-term plateau in the PFS curve, and there is >10% improvement in PFS at 2 years. True (Kindler et al. 2022)

Final, toxicity and QoL adjusted, magnitude of clinical benefit grade	4	3	2	1
	☐	✓	☐	☐

Highest magnitude clinic benefit grade that can be achieved grade 4.

Non-curative setting grading - 5 and 4 indicates a substantial magnitude of clinical benefit

PFS, progression-free survival; OS, overall survival; QoL, quality of life
