# Supplementary material for: Olaparib not cost-effective as maintenance therapy for platinum-sensitive, BRCA1/2 germline-mutated metastatic pancreatic cancer
Source: PLoS One. 2024 Apr 4;19(4):e0301271. doi: 10.1371/journal.pone.0301271 (PMC10994352; doi:10.1371/journal.pone.0301271)
Supplement: Attachment — Submitted filename: Review Report.docx [file pone.0301271.s003.docx]

**Ref. No: PONE-D-23-40742**

I have reviewed the manuscript and found the following areas to be improved in the revised version.

Comment 1: Provide some latest literature in the introduction related to the study.

Comment 2: Mentioned the novelty of the proposed study and gave future recommendations.
Comment 3: The authors should report the mathematical equations of the proposal highlighting all identities.
Comment 4: there are many typos, grammar, and spelling mistakes authors should read the file thoroughly.
Comment 5: It is recommended to provide a Table having all the notations and abbreviations.
