# Supplementary material for: Olaparib not cost-effective as maintenance therapy for platinum-sensitive, BRCA1/2 germline-mutated metastatic pancreatic cancer
Source: PLoS One. 2024 Apr 4;19(4):e0301271. doi: 10.1371/journal.pone.0301271 (PMC10994352; doi:10.1371/journal.pone.0301271)
Supplement: Attachment — Submitted filename: Response_to_Reviewers_PlosOne_V2.0_JL_ms_TM_clean.docx [file pone.0301271.s004.docx]

**Tarun Mehra, M.D.**

Department of Medical Oncology and Hematology

University Hospital Zurich

Rämistrasse 100

CH-8091 Zurich

Mr Qi Chen, PhD

Academic Editor

PLOS One
1265 Battery Street

Suite 200

San Francisco, CA 94111

USA

Zurich, Tueseday 27^th^, Febuary 2024

**Response to Reviewers: “Cost-Effectiveness & Budget Impact Analysis of Olaparib for *BRCA 1/2* Germline-mutated Pancreatic Cancer” - PONE-D-23-40742**

Dear Mr Qi

Many thanks for giving us the opportunity to substantially improve our manuscript.

We reviewed and amended our manuscript to comply with the journal’s style (title, headings, author affiliations, tables, figures and references).

We have an ethics statement in the Methods section. As our study uses published or open-source data, an approval from an ethical review board was not mandatory, in accordance with Swiss regulations. Therefore, it was also not necessary to retrieve specific, individual, per-patient informed consent.

We removed the funding statement from the Acknowledgement section.

We amended the funding statement, which now reads: *“This study was partially funded by the Swiss Study Group for Clinical Cancer Research (SAKK, Schweizerische Arbeitsgemeinschaft für Klinische Krebsforschung). The funder had no role in study design, data collection and analysis, decision to publish, or preparation of the manuscript. There was no additional external funding received for this study.”*

We amended the financial disclosure statement to include the Statement: *“This study was partially funded by Swiss Study Group for Clinical Cancer Research (SAKK, Schweizerische Arbeitsgemeinschaft für Klinische Krebsforschung)."* We also amended the Competing Interests section with the sentences: “*All authors have read the journal's policy and the authors of this manuscript have the following competing interests.”* and *“This does not alter our adherence to PLOS ONE policies on sharing data and materials.”*

We have added captions for our Supporting Information files.

We haven further taken care to respond thoroughly to the Reviewers comments:

Reviewer 1

We thank the Reviewer for taking time to read and review our manuscript. The valuable comments help us to substantially improving its quality.

Comment 1: Provide some latest literature in the introduction related to the study.

- We conducted a new thorough review of the literature and amended the introduction and discussion. In particular, we now reference the studies of Fashami et al. [1] and Ahn et al. [2]

Comment 2: Mentioned the novelty of the proposed study and gave future recommendations.

- We especially thank the Reviewer for pointing out the possibility of highlighting the novelty of our study. We amended the Discussion to this regard. In *Strengths and limitations*, we added the following sentences to the *Strengths and Limitations*: “To our knowledge, our study is the first to consider the screening cost of the entire potentially eligibly population in a cost-effectiveness analysis of olaparib.” and *“Furthermore, to our knowledge, our study is the first to highlight the substantial financial burden of companion diagnostic testing on the budget impact of introducing olaparib in metastatic pancreatic cancer; a finding which should be considered when evaluating other targeted therapies.”*
- We give a related recommendation in the conclusion at the end of the Discussion: *“Olaparib is cleary not a cost-effective treatment option in this patient population at current costs and would have a substantial budget impact. It is important to consider the costs incurred by companion diagnostics when undertaking cost-benefit assessments of targeted therapies in the future.”*

Comment 3: The authors should report the mathematical equations of the proposal highlighting all identities.

- We have added the respective equations in the supplement with a reference in the manuscript, where the partitioned survival model is mentioned for the first time. The new section in the Methods of the Supplementary File 1 now reads:

“A partitioned survival model (PSM) with *N* states calculates the probability patients being in various health states at a specific time during treatment with a particular therapy. State membership is determined by survival curves that do not overlap; in the case of an N-state model, it necessitates *N*-1 survival curves.

The cumulative survival function is

*S_n_*(*t*)

It describes the probability of a patient's survival to health state *n* or to a state with a lower index beyond a specified time *t*. The likelihood of a patient being in health state 1 is represented by *S*_1_*(t)*. Membership in health states 2,…,*n* −1 is determined by the difference between *S_n_*(*t*) and *S_n_*_−1_(*t*). The final health state *N* (when patients are dead) is 1−*S_N_*_−1_*(t*) [3].“

Comment 4: there are many typos, grammar, and spelling mistakes authors should read the file thoroughly.

- We conducted a thorough revision of spelling and grammar.

Comment 5: It is recommended to provide a Table having all the notations and abbreviations.

- We are thankful for this suggestion. The additional table has been included as Supplementary Table S1.

With many thanks and kind regards, on behalf of the co-authors

Tarun Mehra, M.D.

References

1.	Mirzayeh Fashami F, Levine M, Xie F, Blackhouse G, Tarride J-E. Olaparib versus Placebo in Maintenance Treatment of Germline BRCA-Mutated Metastatic Pancreatic Cancer: A Cost–Utility Analysis from the Canadian Public Payer’s Perspective. Current Oncology. 2023;30(5):4688-99. PubMed PMID: doi:10.3390/curroncol30050354.

2.	Ahn ER, Rothe M, Mangat PK, Garrett-Mayer E, Calfa CJ, Alva AS, et al. Olaparib in Patients With Pancreatic Cancer With BRCA1/2 Mutations: Results From the Targeted Agent and Profiling Utilization Registry Study. JCO Precision Oncology. 2024;(8):e2300240. doi: 10.1200/po.23.00240. PubMed PMID: 38354330.

3.	Network TCRA. Partitioned survival models. 2024.
